# Effect of the Reactor Material on the Reforming of Primary Syngas

**DOI:** 10.3390/molecules29215126

**Published:** 2024-10-30

**Authors:** Claudia Bezerra Silva, Michael Lugo-Pimentel, Carlos M. Ceballos, Jean-Michel Lavoie

**Affiliations:** Laboratoire des Technologies de la Biomasse, Département de Génie Chimique et de Génie Biotechnologique, Faculté de Génie, Université de Sherbrooke, Sherbrooke, QC J1K 2R1, Canada; claudia.bezerra.silva@usherbrooke.ca (C.B.S.); m.a.lugo00@gmail.com (M.L.-P.); carlos.mario.ceballos.marin@usherbrooke.ca (C.M.C.)

**Keywords:** syngas, dry reforming, Inconel catalytic activity, stainless steel, carbon dioxide conversion, methane conversion

## Abstract

Syngas, mostly hydrogen and carbon monoxide, has traditionally been produced from coal and natural gas, with biomass gasification later emerging as a renewable process. It is widely used in fuel synthesis through the Fischer–Tropsch (FT) process, where the H_2_/CO ratio is crucial in determining product efficiency and quality. In this sense, this study aimed to reform an emulated syngas resulting from the supercritical water gasification of biomass, tailoring it to meet the H_2_/CO ratio required for FT synthesis. Conditions resembling dry reforming were applied, using temperatures from 600 to 950 °C and steel wool as a catalyst. Additionally, the effects of Inconel and stainless steel as reactor materials on syngas reforming were investigated. When Inconel was used, H_2_/CO ratios ranged between 1.04 and 1.84 with steel wool and 1.28 and 1.67 without. When comparing reactions without steel wool performed either in the Inconel or the stainless steel reactors, those using Inconel consistently outperformed the stainless steel ones, achieving CH_4_ and CO_2_ conversions up to 95% and 76%, respectively, versus 0% and 39% with stainless steel. It was concluded that the Inconel reactor exhibited catalytic properties due to its high nickel content and specific oxides.

## 1. Introduction

Synthesis gas, commonly known as syngas, is a mixture composed of hydrogen (H_2_), carbon monoxide (CO), carbon dioxide (CO_2_), and nitrogen (N_2_), in addition to methane (CH_4_) and other minor components. CO and H_2_ play crucial roles in various chemical processes and energy production [1,2]. Gasification is one of the most common processes for producing syngas, traditionally utilizing coal or natural gas as feedstock, although other carbonaceous materials, such as biomass, can be employed [3]. Biomass is a carbon-neutral feedstock and has been considered through the years for gasification, transforming its low energy density into standard syngas that can be utilized in a wide range of applications, including heat, power generation, and chemical production [4].

Gasification involves numerous technologies and process adaptations. However, with growing interest towards hydrogen production, syngas can also be produced through biomass supercritical water gasification (SCWG). This thermochemical technology uses water, under supercritical conditions (pressure and temperature over 22 MPa and 374 °C, respectively) as the gasifying agent [5,6,7]. The EU H2020-project CERESiS (“Contaminat**E**d land Remediation through Energy crops for Soil improvement to liquid fuel Strategies”) evaluates SCWG as a method for converting energy crops into a hydrogen-rich syngas. Although the first target was the production of hydrogen, this gas was also considered as feedstock for fuel production through the Fischer–Tropsch (FT) synthesis [8,9,10].

Syngas composition, particularly the H_2_/CO ratio, must be carefully tailored to meet the specific requirements of downstream applications. For example, the production of ethanol and higher alcohols typically requires a H_2_/CO ratio of about 1, while for Fischer–Tropsch (FT) synthesis, the primary reaction commonly demands a H_2_/CO ratio of around 2 [11,12]. Some studies suggest that favorable FT conversions can be achieved with lower H_2_/CO ratios, starting at 0.5, when using an iron-based catalyst [13,14,15].

The H_2_ concentrations in the gas produced by SCWG are usually higher than those in conventional gasification processes [16,17]. In this sense, to make syngas produced from SCWG suitable for FT synthesis, it is essential to achieve the appropriate H_2_/CO ratio, which can be reached through hydrocarbon reforming technologies such as dry, steam, and partial oxidation, and autothermal reforming. Each of these technologies uses a different agent, yielding different H_2_/CO ratios [18,19,20]. For example, in dry reforming, CO_2_ reacts with hydrocarbons to produce CO and H_2_ (Equation (1)). When the involved hydrocarbon is methane (CH_4_), its reaction with CO_2_ usually leads to a H_2_/CO ratio of 1 (Equation (2)) [21].

(1) $$C_{n}H_{m}+nCO_{2}↔2nCO+{(n+1)H}_{2}$$

(2) $$CH_{4}+CO_{2}↔2CO+2H_{2}\;(+247\;\mathrm{kJ}/\mathrm{mol})$$

Dry reforming is a highly endothermic process that demands significant energy. It typically operates at temperatures between 600 and 1000 °C [22,23]. Optimal conversion of methane and CO_2_ is generally reported to be achieved in the narrower range of 750–900 °C, where syngas with a high hydrogen-to-carbon monoxide ratio can be produced [24]. The gas produced by this technique leads to the formation of chemicals and liquid hydrocarbons when used in Fisher–Tropsch synthesis. Nevertheless, it is also possible that some side reactions occur in addition to the primary reaction shown in Equation (2), which will, in turn, decrease the H_2_/CO ratio. These side reactions include reverse water–gas shift (RWGS), CH_4_ decomposition, disproportionation of CO, and hydrogenation of CO_2_ and CO reactions (Equations (3)–(7)) [21]. At lower temperature (closer to 600 °C) the reverse water–gas shift (RWGS) reaction becomes more dominant, while at temperatures above 900 °C, the risk of carbon deposition increases due to methane and CO_2_ cracking, leading to rapid catalyst deactivation [25].

(3) $$CO_{2}{+H}_{2}↔CO+H_{2}O\;(+41\;\mathrm{kJ}/\mathrm{mol})$$

(4) $$CH_{4}↔C+2H_{2}\;(+75\;\mathrm{kJ}/\mathrm{mol})$$

(5) $$2CO↔C+CO_{2}\;(-172\;\mathrm{kJ}/\mathrm{mol})$$

(6) $$CO_{2}+2H_{2}↔C+2H_{2}O\;(-90\;\mathrm{kJ}/\mathrm{mol})$$

(7) $$H_{2}+CO↔H_{2}O+C\;(-131\;\mathrm{kJ}/\mathrm{mol})$$

Catalysts are essential in the dry reforming process since they might decrease the reaction activation energy, which can reduce the amount of energy necessary for the process while increasing conversion to syngas [26]. In this sense, the catalyst chosen for dry reforming should be stable and, simultaneously, highly coke-resistant. Numerous studies have been conducted in this field for various supported metals and noble metal catalysts such as rhodium, ruthenium, palladium, platinum, and iridium [27]. Research is being conducted to find alternatives to noble metals due to their high cost, with substitutes like nickel and cobalt being explored. Among these, nickel-based catalysts are widely recognized and extensively studied for the dry reforming of methane (DRM). These catalysts are known for their high conversion rates of CO_2_ and CH_4_ and their efficiency at lower temperatures. However, a significant issue with Ni-based catalysts is carbon deposition, leading to deactivation [28,29]. One of the strategies used to decrease carbon formation and increase catalytic stability is to use an adequate promoter in the Ni-supported catalyst. Iron metal could act as a good promoter due to its ability to increase oxygen mobility and inhibit carbon deposition [30]. Moreover, the iron redox property was shown to influence the DRM mechanism [31]. Bian et al. [32] reviewed nickel-based catalysts for the dry reforming of methane, and in this work, the redox properties of some Ni-Fe catalysts are shown. For all the catalysts mentioned, the DRM’s carbon resistance was improved. This was probably due to CO_2_ oxidation and CH_4_ reduction mechanisms. In this context, steel wool, primarily composed of iron (98.5%) [18], has been studied as a catalyst for the dry reforming of methane. Research by Labrecque and Lavoie [33] and Banville et al. [18,34] have shown that using steel wool as a catalyst can positively impact methane conversion in the DRM process. Additionally, steel wool is widely available, cost-effective, and offers a large surface area, making it an appealing option as catalyst [35].

Another critical aspect is the influence of the reactor material on the catalytic process. Various studies have shown that reactor wall materials can significantly impact reaction outcomes. Salierno et al. [36], when comparing stainless steel-316 and Inconel-625, showed that high nickel content in Inconel-625 could raise hydrogen consumption pathways due to the rise in methane production at the expense of hydrogen yield, while increasing C_2_ hydrocarbon production. Tuan Abdullah and Croiset [37] assessed ethanol reforming within an Inconel-625 reactor, demonstrating that a larger surface-to-volume reactor ratio increased ethanol conversion. Bustamante-Londono [38] reported improved performance using an Inconel reactor over quartz reactors in the water–gas shift reaction; it demonstrated high conversion improvements under higher temperature and pressure conditions. Li [39] and Boukis and Habicht et al. [40] researched the corrosion behavior of Inconel 625 by SCWG, referring to a strong interaction with reaction products and indicating the dominating mechanisms of corrosion. According to Zhu et al. [41], nickel has a catalytic function in raising the efficiency of glycerol and glucose gasification. These studies appear with a new emphasis on the critical role that reactor wall materials can play in determining catalytic activity, conversion rates, and product distribution.

In this sense, this study seeks to optimize an emulated gas composition that mimics the output from supercritical water gasification, tailoring it to meet Fischer–Tropsch synthesis requirements. Specifically, this study focused on adjusting the H_2_/CO ratio through conditions originally tailored for dry reforming. The reactions were performed in a fixed bed Inconel reactor with temperatures ranging from 600 to 950 °C, employing steel wool as a catalyst for the catalyzed reactions. Temperatures between 600 and 950 °C were used because this range involves critical thermal conditions where the DRM reaction becomes efficient. Below 600 °C, the reaction rate is normally slow, and catalyst deactivation due to coking is also more substantial. Meanwhile, temperatures beyond 950 °C raise the risk of sintering into the catalyst, hence degrading the performance. The study also investigated the effects of the interaction between the reactor wall materials—Inconel and stainless steel—and the gaseous reaction medium on methane and carbon dioxide conversions during the reactions.

## 2. Results and Discussion

### 2.1. Comparison of Experiments With and Without Steel Wool (SW) Using an Inconel Reactor

**Figure 1:**
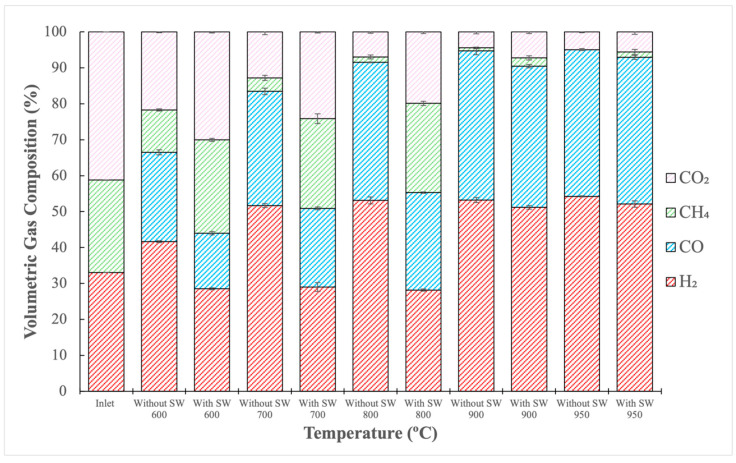
Volumetric gas composition of reformed gas in Inconel reactor (600–950 °C, with and without steel wool).

The emulated gas was subjected to a reforming reaction in an Inconel fixed bed reactor at temperatures ranging from 600 to 950 °C, with and without steel wool as a catalyst. Gas composition after each reforming experiment is shown in Figure 1, where the first bar on the graph (named inlet) corresponds to the inlet gas composition before each reforming reaction.

Analyzing gas composition in Figure 1 revealed some critical information. Initially, the amounts of H_2_ and CO for each temperature tested increased for the reactions performed without steel wool. In contrast, the amounts of CH_4_ and CO_2_ decreased. Two potential explanations for these findings can be considered. Steel wool may have had a detrimental effect on the reaction by increasing iron content [42], or the reactor’s material might have acted as a catalyst [36,37,38,39,41].

The H_2_/CO ratio for each tested condition was calculated using gas composition after the reforming reaction, by dividing the volumetric composition of H_2_ by that of CO. Furthermore, the amount of water produced during reactions is another critical parameter to evaluate for a more in-depth analysis (see Table 1).

The first observation from Table 1 is that, for the experiments conducted without SW, both water production and H_2_/CO ratio follow a decreasing trend as temperature increases. At lower temperatures, the RWGS reaction (Equation (3)) was likely favored, resulting in increased water production. However, an increase in the H_2_/CO ratio was also observed at lower temperatures. This raises some confusion when associating the rise in water solely to the reverse water–gas shift (RWGS) reaction. In the RWGS reaction, water production occurs alongside an increase in CO. Therefore, if CO increased, the H_2_/CO ratio should have decreased, not increased, as the results suggest. It can be assumed that a kinetic phenomenon caused a decrease in CO while the amount of water increased, which could explain this discrepancy. However, caution should be exercised as it cannot be concluded that RWGS is the only reaction occurring under these conditions. Other reactions could have contributed to the observed results, such as CH_4_ decomposition (Equation (4)), which produces more H_2_, or the disproportionation of CO (Equation (5)), which consumes some of the CO generated by the RWGS. These processes could have led to a higher H_2_/CO ratio. Ultimately, these findings suggest the need for further investigation and the potential for discoveries in this area. In contrast, no clear trend was observed when reactions were performed using SW. However, when comparing the results for each temperature, with and without SW, it was noted that water production was higher at 600 and 700 °C without SW. In contrast, at higher temperatures (800 to 950 °C), the opposite trend happened, with more water being produced in the presence of SW. This might be related to the iron content in the wool. There is some evidence that when the RWGS reaction is processed at high temperatures, iron could act as a thermal stabilizer for the process [43]. For instance, research by Chen et al. [44] has demonstrated that incorporating iron into a Cu/SiO_2_ catalyst enhances both the stability and activity of the catalyst during the RWGS process, resulting in improved performance over time.

At 950 °C, the H_2_/CO ratio was not significantly affected by the presence or absence of steel wool (SW). This outcome can be attributed to the likelihood that certain reactions, such as methane dry reforming (Equation (2)), may have reached the equilibrium at such elevated temperatures, thereby similarly influencing the production of CO and H_2_. Additionally, water production decreased substantially in the absence of SW, suggesting that SW may act more effectively as a catalyst for the reverse water–gas shift (RWGS) reaction. Without SW, the RWGS reaction appears to be less active, resulting in reduced water formation at this temperature. In contrast, methane reforming persists as the dominant process, maintaining a constant H_2_/CO ratio.

While the H_2_/CO ratio is essential in determining whether syngas is suitable for a given application, it cannot be the sole deciding parameter. The concentration of other hydrocarbons in the gas must also be considered. Table 1 shows that a higher H_2_/CO ratio was obtained at 600 °C when using SW. However, Figure 1 shows that, under these conditions, the reformed syngas contained higher amounts of unconverted CH_4_ and CO_2_ than in all other experiments. Thus, the best outcome was achieved at 950 °C without SW, which resulted in less unreacted CH_4_ and CO_2_ with a H_2_/CO ratio of 1.33. Generally, Fischer–Tropsch synthesis requires a H_2_/CO ratio of about 2. However, when using iron-based catalysts, ratios as low as 0.5 are feasible [13,14,15]. Thus, the H_2_/CO ratio achieved during these experiments could fit in the acceptable range for Fischer–Tropsch synthesis.

**Figure 2:**
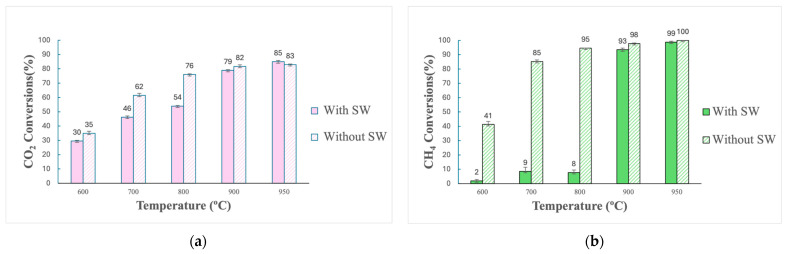
Comparison of CO_2_ (**a**) and CH_4_ (**b**) conversions after reforming in Inconel reactor (600–950 °C, with and without steel wool).

When syngas reforming is performed, the goal is to improve the H_2_/CO ratio in the gas while ensuring that CH_4_ and CO_2_ react (as in Equation (2)). Therefore, another measure of process efficiency is the conversion of CH_4_ and CO_2_ (Figure 2).

Figure 2 shows that the conversion rates of CH_4_ and CO_2_ correspondingly increase as temperature rises. This outcome was expected due to the nature of dry reforming, which is a highly endothermic reaction that is favored at elevated temperatures. Higher temperatures enhance the likelihood of CH_4_ and CO_2_ reacting to form CO and H_2_ [29]. As a result, it is unsurprising that the highest conversions of CH_4_ and CO_2_ were observed at 950 °C among all tested temperatures. Additionally, Figure 2 shows that CH_4_ conversion was higher compared to CO_2_. This can be explained by the fact that the gas fed into the experiment had a CH_4_/CO_2_ ratio of 0.62, below the stoichiometric dry reforming ratio of 1. This means that CO_2_ was fed in a greater amount than CH_4_. Consequently, CH_4_ reacted with a proportional amount of CO_2_, leaving a higher proportion of unreacted CO_2_. An important observation is that, for temperatures between 600 and 800 °C, steel wool seems to have had a more pronounced negative effect on CH_4_ conversions than on those of CO_2_. For example, at a temperature of 600 °C, with steel wool, the CH_4_ conversion obtained was 2%, while without it, the conversion increased to 41%. At this same temperature, the CO_2_ conversion obtained with steel wool was 30% and increased to 35% without it. Therefore, the increase in conversion was more significant for CH_4_ than for CO_2_.

Methane conversion requires breaking the C-H bond, a process that typically occurs at high temperatures [45]. The observed decrease in CH_4_ conversion at lower temperatures may result from the inhibition of surface interactions needed for C-H bond cleavage on steel wool. This could be due to the reduced capacity of the surface to facilitate dissociation under these conditions. Catalysts, particularly transition metals like cobalt, iron, nickel, and copper, are often necessary for methane decomposition at lower temperatures [45]. Since steel wool consists primarily of iron, it likely influences the reaction, while in its absence, the Inconel reactor—composed mainly of nickel—provides the active surface. Literature suggests that iron is more efficient for methane decomposition at higher temperatures, supporting the idea that steel wool inhibits this process at lower temperatures [46]. Once the steel wool was removed, it could have led to a more noticeable increase in CH_4_ conversion. However, the mechanism by which this process occurred is unknown.

Although nickel is the primary component of the Inconel alloy, it is important to remember that numerous other metals are also present [8]. The interaction of the reactor walls with steel wool, primarily composed of iron but containing traces of other elements, could follow several pathways. Some of these elements might decrease activity, potentially hindering the formation of certain species. The interaction between one or more reactor components and steel wool could have created an effect that ultimately inhibited CH_4_ conversion.

Notably, in the results presented in Figure 2, nearly all reactions without steel wool achieved higher CH_4_ and CO_2_ conversions. The only case where these conversions were not higher without steel wool was at 950 °C, where CO_2_ conversion went from 85 to 83% when the reaction went from using steel wool to not using it. This represents a percentage difference of 2.43%, which was not considered a significant decrease in this case. Manabayeva et al. [42] investigated the effects caused from varying the loads of Fe on a nickel–alumina (Ni-Al) catalyst for CH_4_ and CO_2_ conversions. Results indicated that higher amounts of iron negatively impacted the catalytic efficiency of the process. It was demonstrated that increasing iron content resulted in larger catalytic particle sizes, which reduced the available surface area and, thus, decreased catalytic activity. Several other hypotheses were suggested to explain the decrease in CH_4_ and CO_2_ conversions with higher Fe loads. One possible explanation is that the catalyst’s capability to absorb hydrogen may decrease. Additionally, adding more Fe might not ensure the proper formation of a Ni-Fe alloy since excess Fe could migrate to the catalyst surface, reducing the exposure of the Ni-Fe alloy to the reaction and decreasing its activity.

### 2.2. Effects of CH_4_/CO_2_ Ratio on Reforming Reaction

Additional tests were performed to evaluate the influence of different gas compositions in reforming reactions. All these tests were performed considering the operation conditions that resulted in the best outcome (Section 2.1): at 950 °C, using an Inconel reactor and in the absence of steel wool. Table 2 illustrates the behavior of CH_4_ and CO_2_ conversions to the CH_4_/CO_2_ ratio used in the process.

The results shown in Table 2 were somehow expected since the gas containing the lowest CH_4_/CO_2_ ratio (gas 3, CH_4_/CO_2_ = 0.47) had the smallest CO_2_ conversion. The composition of the third gas fed into the process (#3) contained a higher amount of CO_2_ as compared to the other gases. Hence, this could explain the greater the amount of unconverted CO_2_. If the dry reforming reaction ((Equation (2)) is assumed to be the primary reaction occurring, CH_4_ and CO_2_ should react at a 1:1 molar ratio (CH_4_/CO_2_ = 1). When the CH_4_/CO_2_ ratio is less than 1, it means that proportionally, more CO_2_ than CH_4_ is available, implying that the CO_2_ conversion will be limited by the amount of CH_4_. Then, for a process where CH_4_/CO_2_ < 1, the greater the amount of CO_2_ fed will also mean more unconverted CO_2_ thus decreasing its overall conversion. Noteworthy that the conversion of CO_2_ might not be only related to the reaction with CH_4_, as shown in Equation (2). Since 33% of H_2_ was also fed into the process, reactions involving H_2_ and CO_2_, such as the reverse water–gas shift, might also have occurred.

The effect of having a CH_4_/CO_2_ ratio lower than 1 has also been investigated in several studies. For example, Han et al. [47] found that using a CH_4_/CO_2_ feed ratio of 0.69 on a NiCeO_x_ catalyst at 700 °C resulted in CH_4_ and CO_2_ conversions of 90.6% and 60.2%, respectively. Similarly, Khajenoori et al. [48] reported a CH_4_/CO_2_ ratio of 0.67 and a NiCeO_2_/MgO catalyst at 700 °C, achieving CH_4_ and CO_2_ conversions of approximately 75% and 45%, respectively. Moreover, considering the interaction between CH_4_ and CO_2_ in conventional dry reforming (shown in Equation (2)), the reaction requires the dissociation of the C-H bonds in CH_4_ and the C=O bonds in CO_2_ molecules, respectively [49]. For instance, C-H bond breakage requires about 439 kJ mol^−1^ [50], while C=O requires 799 kJ mol^−1^. Therefore, more energy might be necessary to convert CO_2_ than CH_4_ [51]. Studying different gas mixtures is hence essential in order to understand the effectiveness and dynamics of the dry reforming process. Furthermore, changes in the CH_4_/CO_2_ ratio are known to show the impact of the conversion of the reactants and the selectivity of the products. Khajenoori et al. [48], when studying dry reforming with Ni–CeO_2_/MgO catalysts, observed that decreasing the CH_4_/CO_2_ ratio from 4 to 2 increased CH_4_ and decreased CO_2_ conversions. Osazuwa and Cheng [52] investigated different CH_4_/CO_2_ ratios at 750 °C for CH_4_/CO_2_ = 2. In such situation, CH_4_ was converted at 66% whereas for CH_4_/CO_2_ = 1, the conversion was of 84%. Besides the difference in CH_4_ conversion, the H_2_ produced increased from 45% to 60% when the CH_4_/CO_2_ changed from 2 to 1. In a study performed by Zhang et al. [53], besides conventional conversions, product selectivity was investigated when varying the CH_4_/CO_2_ ratio from 3:1 to 1:3. The variation increased CH_4_ and decreased CO_2_ conversions. The excess of CO_2_ influenced a more pronounced reaction between CO_2_ and H_2_, thus increasing by-products and decreasing the H_2_/CO ratio.

### 2.3. Comparison of Inconel and Stainless Steel Reactors Without Steel Wool

**Figure 3:**
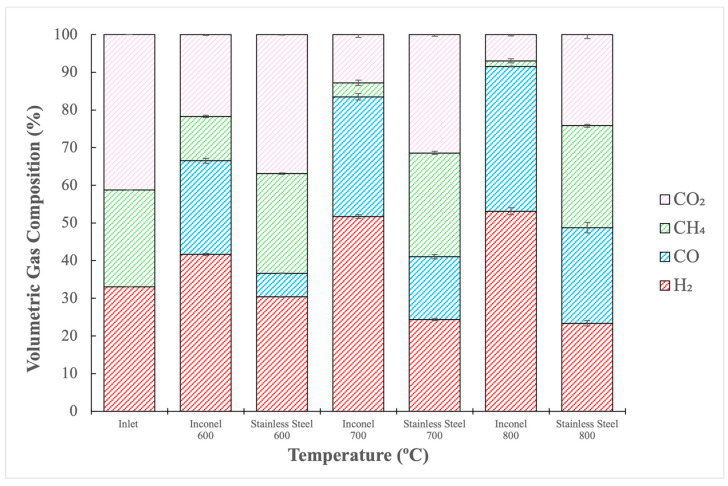
Volumetric gas composition of reformed gas in Inconel and stainless steel reactors at 600–800 °C, without steel wool.

To evaluate the influence of the reactor material on the process, experiments were conducted in a stainless steel (SS) reactor, without steel wool, under the same conditions as the reactions using the Inconel reactor. The results of this investigation are shown in Figure 3, which compares gas compositions after reforming, both in the Inconel and SS reactors, at temperatures ranging from 600 to 800 °C.

As observed in Figure 3, the amounts of H_2_ and CO were higher, while those of CH_4_ and CO_2_ were lower when Inconel was used instead of stainless steel. This suggests that the potentially catalytic walls of the Inconel reactor interacted with the gas phase reactions, enhancing reaction pathways to increase consumption of CH_4_ and CO_2_ along with the increase in H_2_ and CO production.

To expand on these results, Table 3 compares the H_2_/CO ratio and water produced per hour for Inconel and stainless steel reactors at all tested temperatures.

In a conventional dry reforming process, no H_2_ would be found in the inlet, and the H_2_/CO ratio would be a good indication of the reaction’s efficiency. A high-performing conventional dry reforming process typically achieves high methane conversion and a H_2_/CO ratio of 1 [28]. This means that the reaction between CO_2_ and CH_4_ (Equation (2)) was possibly the main one to occur in the process and that side reactions, involving the consumption of H_2_ and CO, were not facilitated. In this work, a considerable amount of H_2_ was already fed into the reaction, hence, a H_2_/CO ratio of 1 cannot necessarily indicate that the main reaction was dry-reforming of CH_4_ (Equation (2)). However, if the reaction produced a H_2_/CO ratio of less than 1 and the H_2_ amount decreased compared to the inlet, it would have been a good sign that H_2_ was consumed by side reactions.

That was the case for the reaction in the SS reactor at 800 °C. Table 3 shows a H_2_/CO ratio of 0.92, with 4.39 g/h of water produced, while Figure 3 shows that the reformed gas had a H_2_ concentration lower than the inlet. By comparing the results at the same temperature, however, an H_2_/CO ratio of 1.38 was obtained when the Inconel reactor was used, and 1.85 g/h of water was produced, leading to a reformed gas with a higher H_2_ content than that of the inlet. This could mean that, when operating in a stainless-steel reactor, H_2_ was consumed to produce water following a reverse water–gas shift reaction (Equation (3)). Inconel, on the other hand, could have enhanced the dry-reforming reaction at this temperature, leading to a higher amount of CO and H_2_ while producing less water through a reverse water–gas shift reaction.

In Table 3, at 600 °C, the H_2_/CO ratio difference between both reactors was high: 1.67 and 4.86, for Inconel and SS, respectively. After a deep analysis of the volumetric compositions presented in Figure 3 for the operation at 600 °C, it can be observed that the gas inlet fed in the reactions presented 33.05% H_2_ and no CO. After reforming with the Inconel reactor, the gas presented 41.63% H_2_ and 24.88% CO, while for stainless steel, the H_2_ content was 30.4% and that of CO was 6.25%. Thus, a significantly smaller amount of CO was produced in the stainless-steel reactor compared to the Inconel reactor, leading to a substantial difference in the H_2_/CO ratio. Additionally, at 600 °C, experiments conducted with the Inconel reactor generated more water than those with stainless steel. These significant differences in the H_2_/CO ratio and water production can be attributed to the catalytic activity of the Inconel reactor, which likely enhanced the rate of the reverse water–gas shift (RWGS) reaction (Equation (3)), resulting in higher CO and H_2_O production, inversely affecting the H_2_/CO ratio when compared to stainless steel. The results suggest that, unlike Inconel, stainless steel lacks catalytically active metals that promote H_2_ and CO production. Furthermore, analyzing the results from Figure 3 at 600 °C, and considering the concentrations of CH_4_ and CO_2_ in the reformed gas, the Inconel reactor exhibited significantly lower CH_4_ content as compared to CO_2_. This suggests that CH_4_ decomposition (Equation (4)) may have occurred, consuming more CH_4_ and generating additional H_2_. If dry reforming had been the sole reaction, the amounts of CH_4_ and CO_2_ would have been more comparable. This would explain why the amount of H_2_ produced increased as compared to the inlet when Inconel was used. Although methane’s decomposition typically occurs around 900 °C, the catalytic decomposition of methane in a Ni-catalyzed DRM reaction begins at approximately 550 °C [54]. This would explain why the methane decomposition reaction can be considered in the temperature range tested (600 to 800 °C), since Ni is the primary compound in the Inconel alloy.

**Figure 4:**
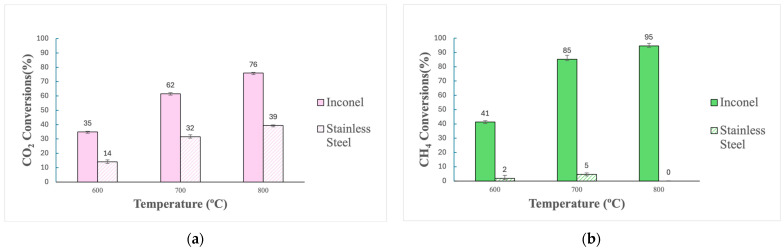
Comparison of CO_2_ (**a**) and CH_4_ (**b**) conversions after reforming in Inconel and stainless-steel reactors (600–800 °C, without steel wool).

At 700 °C, the H_2_/CO ratio and the water produced using the two reactors were comparable. However, using the Inconel reaction led to less CH_4_ and CO_2_ in the reformed gas, making the process more efficient than with the SS reactor. For both reactions at 700 °C, the reverse water–gas shift was believed to have occurred since water was produced in similar proportions. A reverse water–gas shift reaction, for instance, according to the thermodynamic equilibrium, requires temperatures around 600 to 800 °C to occur [55,56]. However, considering the higher amount of CH_4_ and CO_2_ unconverted in the SS reactor and the lower amount of H_2_ and CO produced, it seems that the targeted dry reforming reaction was facilitated by the Inconel reactor. Both, CH_4_ and CO_2_ conversions are reported in Figure 4.

**Figure 5:**
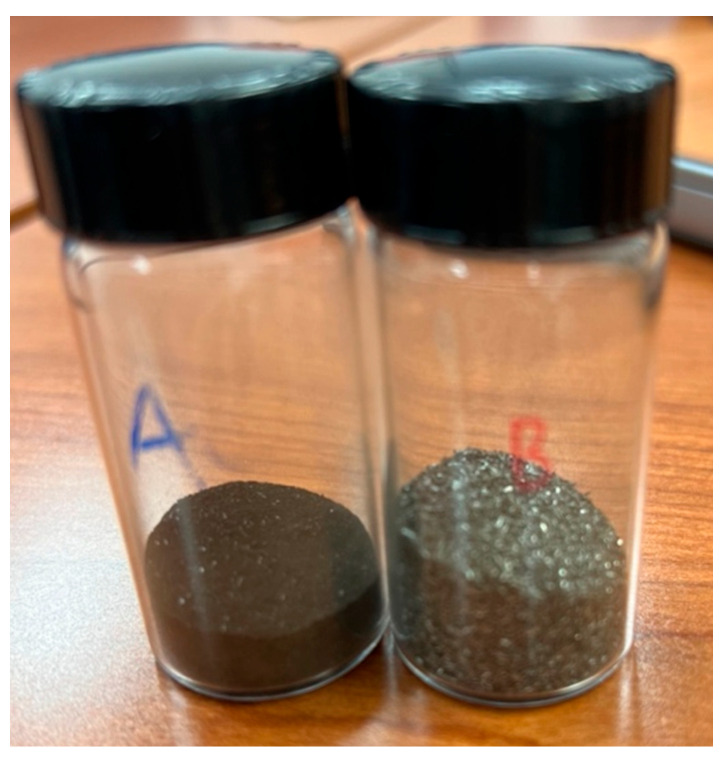
Samples from the (A) innermost and (B) outermost layers from the Inconel reactor used in the reactions.

Figure 4 shows that CH_4_ and CO_2_ conversions were consistently higher with the Inconel reactor than with the SS reactor. Samples from both reactors were thus taken and subjected to XRD and EDS analyses to investigate why reactions in the Inconel reactor presented higher conversions than with stainless steel. An EDS analysis was essential in identifying the elemental composition of the reactors. Identifying different elements that might present catalytic behaviors in the samples from the Inconel reactor might explain why reforming presented much higher conversions in this reactor than with stainless steel. In addition, XRD analyses were used to identify which phases occurring on the reactor’s surface might have contributed to the conversions observed. Two samples from the Inconel reactor were taken, one from inside the reactor (innermost surface layer), where the gas are in contact with the metal, and one from the reactor’s exterior (outermost surface layer). A drill was used to scrape off the material for both surfaces, generating a powder that was used for characterization (see Figure 5).

Figure 5 shows a noticeable visual disparity between the two reactor sections. While sample B resembles a metallic form of Inconel alloy, sample A presents a stark contrast, appearing black in color. This visual dissimilarity is likely linked to the inner portion of the reactor being more exposed to the reaction medium after several experiments, potentially altering the characteristics of the alloy.

**Figure 6:**
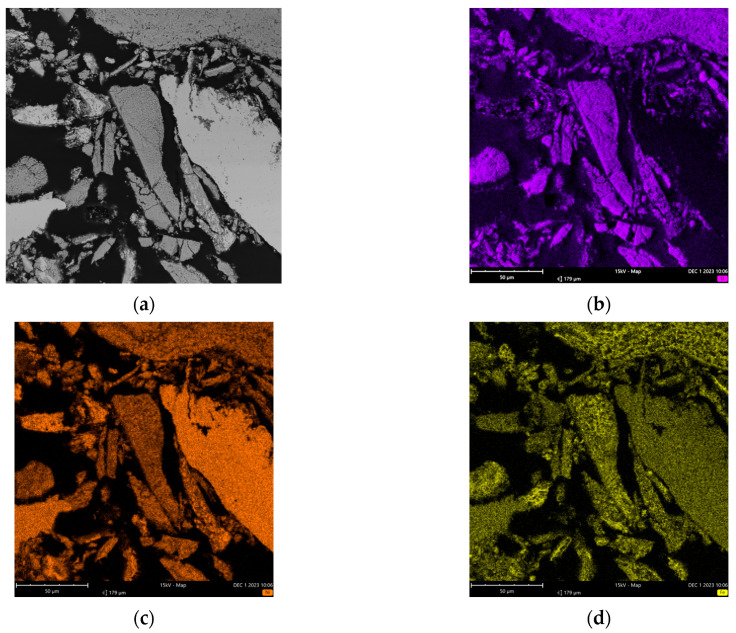

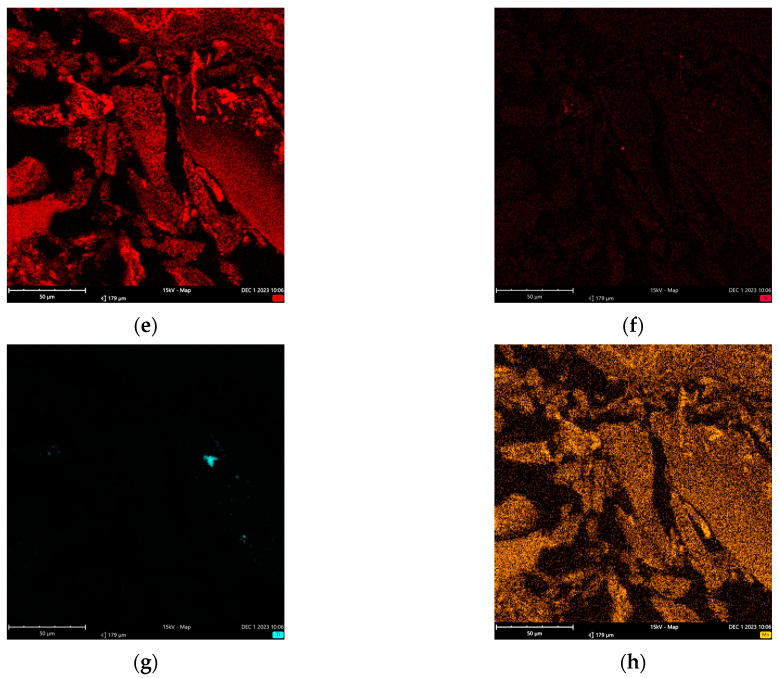
Combined map generated by sample A analysis from the Inconel reactor used in the reactions (FOV: 963 µm; mode: 15 kV-Map; detector: BSD Full): (**a**) SEM image zone; (**b**–**h**) imaging of oxygen, nickel, iron, chromium, silicon, titanium, and manganese.

Figure 6 displays the combined map images from the EDS analysis of sample A from the Inconel reactor.

Figure 6 shows oxygen distributed over nickel particles in addition to smaller amounts of other elements that were also identified, such as chromium, iron, silicon, and manganese. More specifically, Table 4 shows the elemental weight concentration (%) calculated by the EDS analysis for sample A.

**Figure 7:**
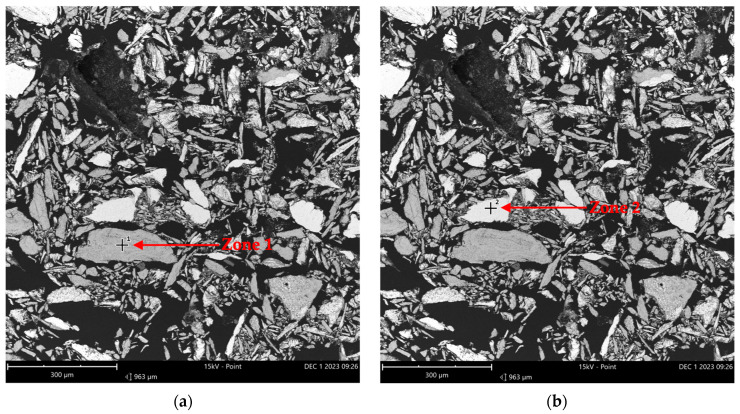
EDS analysis imaging in two zones from sample A (from Inconel reactor used in reactions) (FOV: 963 µm; mode: 15 kV-Point; detector: BSD Full): (**a**) Zone 1 and (**b**) Zone 2 indicate the specific regions chosen for the elemental composition analysis.

The presence of oxygen in Table 4 suggests the potential oxidation of the metals in the Inconel alloy. The use of this reactor in several past experiments, mostly using oxidizing reagents and high temperatures, may have caused the formation of metallic oxides. Table 4 presents the elemental composition for the entire analyzed portion, quantifying all the elements shown in Figure 6. Within this sample, two specific zones (indicated by red arrows in Figure 7) were selected and quantified separately, as detailed in Table 5.

Figure 7 shows that the two chosen zones from sample A exhibit two distinct color patterns. Zone 1 in part (a) appears darker than zone 2 in part (b). These color differences suggest the possibility of varying elements between the two points. Based on the elemental analysis for zone 1, presented in Table 5, it is evident that oxygen and nickel were the predominant elements in this section, indicating the presence of nickel oxide. However, in zone 2, no oxygen was detected, with nickel, chromium, and iron being the major elements. The results from Table 4 and Table 5 suggest that sample A primarily consisted of nickel oxide, pure nickel, and smaller amounts of other metals like chromium and iron, which may or may not be oxidized.

**Figure 8:**
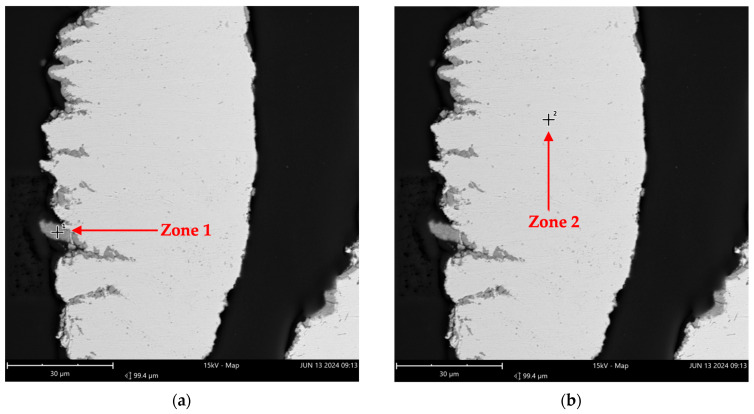
EDS analysis imaging in two zones from sample B (from Inconel reactor used in reactions) (FOV: 99.4 µm; mode: 15kV-Map; detector: BSD Full): (**a**) Zone 1 and (**b**) Zone 2 indicate the specific regions chosen for the elemental composition analysis.

Similarly, sample B was submitted to EDS analysis. Figure 8 shows the imaging, and Table 6 provides the results of the elemental analysis distribution of the two zones pointed out by red arrows.

Figure 8 illustrates that zone 2 appears darker than zone 1. The elemental distribution for zone 1, presented in Table 6, shows that oxygen and chromium were the predominant elements, indicating the presence of chromium oxide. In contrast, zone 2 shows no oxygen, with a higher nickel concentration. Therefore, the analysis in Table 6 suggests that the analyzed portion of sample B primarily consisted of chromium oxide, pure nickel, and other metals.

**Figure 9:**
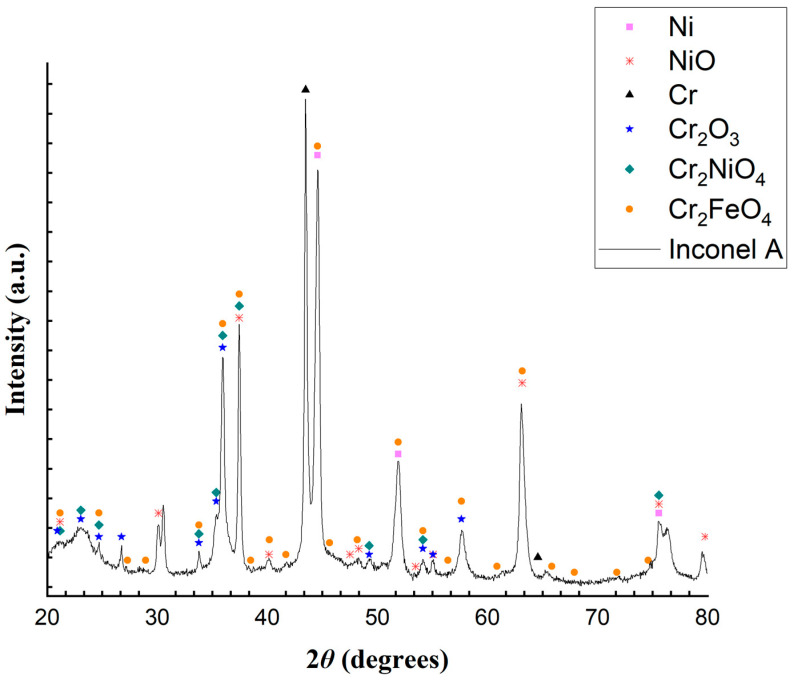
Diffractogram from a powder sample from the Inconel reactor inner layer (sample A).

**Figure 10:**
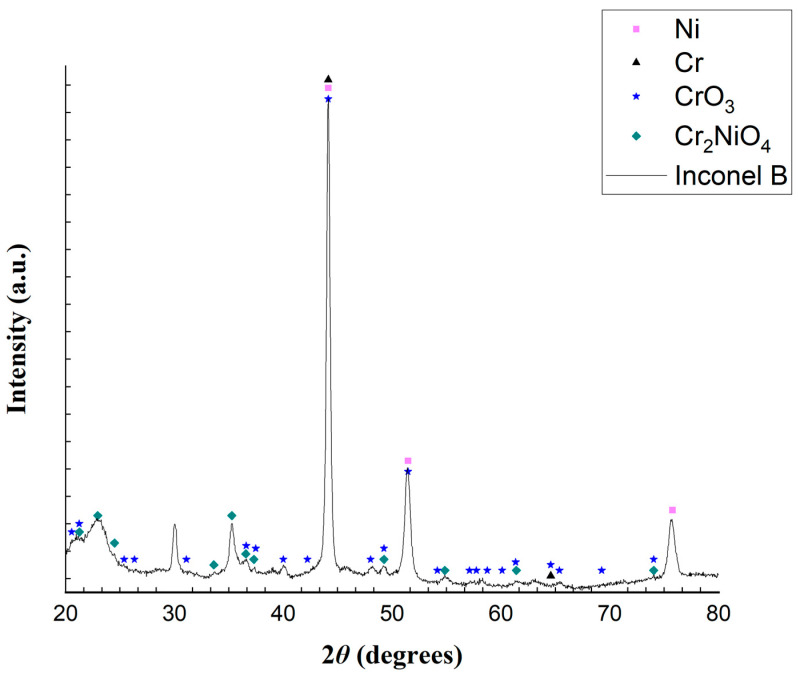
Diffractogram from a powder sample from the Inconel reactor outermost layer (sample B).

An important observation regarding the results presented from the analyses of samples A and B from the Inconel reactor is that, even if the elementary analyses were carried out at specific points in the sample, which may or may not be representative of the reactor, these analyses serve as a way of understanding the possible oxides present in the reactor. In addition, both samples A and B were analyzed by XRD, and the results are shown in Figure 9 and Figure 10.

The XRD patterns in Figure 9 and Figure 10 illustrate the distinctions between the reactor’s inner and outer layers, respectively. The XRD analysis in Figure 10 reveals that sample B contains Ni, Cr, CrO_3_ and Cr_2_NiO_4_. Similarly, the analysis in Figure 9 identifies the presence of Ni, NiO, Cr, Cr_2_O_3_, Cr_2_NiO_4_, and Cr_2_FeO_4_. The outermost layer exhibits fewer peaks than the inner layer, probably because it was less exposed to the reaction medium. The XRD patterns shown in Figure 9, sampled from the reactor used in the experiments, show enough evidence that the Inconel alloy suffered from oxidation. Guo et al. [57], when studying supercritical water gasification, showed that the metal compounds present in alloys such as Inconel 625 can be oxidized and become cations, moving along (by a diffusion process) from the alloy’s interior to its surface, reacting with the oxygen (from the water, for example) and producing oxides and hydroxides that precipitate on the surface of the alloy (Equations (8) and (9)). These products can also react in several ways, producing other metal oxides.

(8) $$M\to M^{n+}+ne^{-}$$

(9) xMS+y2O2→MxOy(S)

It thus seems possible that the reactor used in this work suffered from an oxidative process, as shown in Equations (8) and (9). Table 7 shows possible reactions that could have produced the oxides found in the XRD analysis.

Equations (10)–(13) present reactions showing how Ni, Cr, and Fe can be oxidized to their cationic form. All reactions in Equations (14)–(30) show different pathways leading to the oxides.

**Figure 11:**
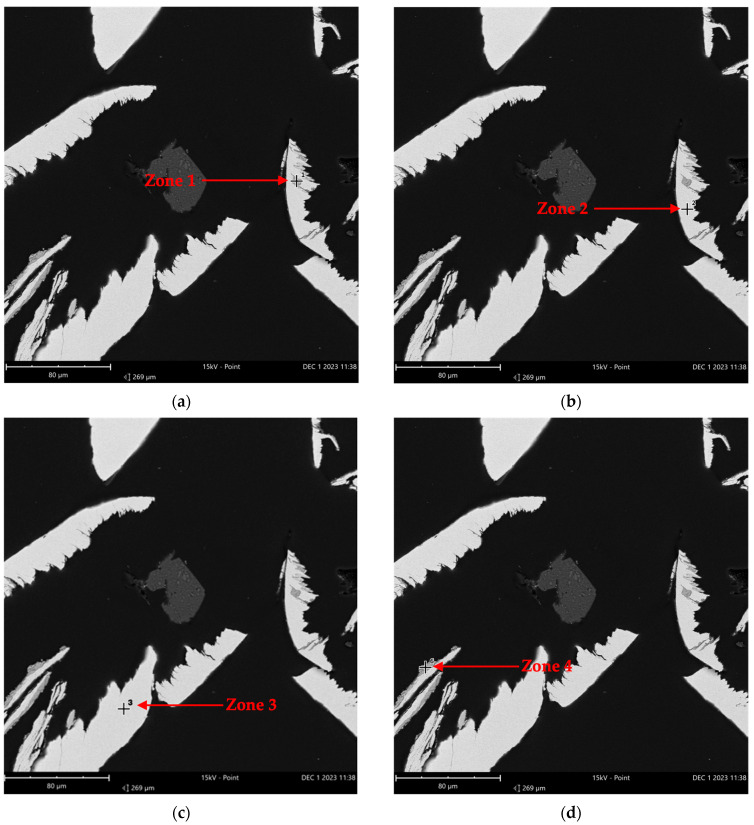
EDS analysis imaging in 4 zones from a sample of the SS reactor used in the reactions (FOV: 269 µm; mode: 15 kV-Point; detector: BSD Full): (**a**–**d**) Zones 1–4 indicate the specific regions chosen for the elemental composition analysis.

The analyses made of the Inconel reactor samples were crucial to explain how the presence of oxides might contribute to the catalytic activity of the Inconel reactor. As a comparison, a sample from inside the stainless-steel reactor was also taken (following the same protocol as for the Inconel reactor samples) and analyzed using EDS. The imaging generated by EDS is shown in Figure 11, and Table 8 shows the results of the elemental analysis distribution for each of the four zones marked by red arrows.

As expected, Table 8 shows that the amount of nickel is much smaller as compared to that in the Inconel reactor and that iron is the most abundant element across the zones. The elemental distribution for zones 1 and 4 shows that oxygen and iron are the predominant elements, indicating the presence of iron oxides. In contrast, for zones 2 and 3, iron remains the major element and chromium replaces oxygen, which is no longer in high concentration such as it was in zones 1 and 4. By comparing the EDS results from Inconel and stainless steel, it becomes clear that the primary difference between the two reactors is the amount of nickel. Although the SS reactor might also contain the same oxides as the Inconel reactor (due to its exposure to several oxidative reactions), the smaller amount of nickel in the SS reactor suggests a different distribution of oxides. Notably, Inconel 625 alloy primarily consists of approximately 60% nickel and 20% chromium [58,59,60]. In this sense, nickel is a significant component of this alloy and exhibits substantial activity in catalyzing the dry reforming of methane reactions. In a study by Salierno et al. [36], the performance of glycerol supercritical water gasification was evaluated in two reactors made from different alloys, namely stainless steel 316 and Inconel 625. When comparing the results of Inconel 625 and stainless steel 316, it is apparent that C_2_ hydrocarbon increased when using the former. The high nickel content in this reactor has been linked to the conversion of an intermediate compound, acetaldehyde, into hydrocarbons (like C_2_ hydrocarbons) and carbon monoxide. Moreover, the chromium content might have helped promote disproportionation reactions. Studies specifically focused on the use of chromium in dry reforming are relatively limited. In the available literature, chromium is often utilized as a promoter in bimetallic catalysts, though there are fewer works addressing this compared to other elements. For instance, Babakouhi et al. [61] investigated a Ni/Al_2_O_3_-CeO_2_ catalyst for the combined CO_2_ reforming and partial oxidation of methane and found that incorporating up to 3% chromium led to a notable improvement in the catalyst’s performance. With the addition of chromium, CH_4_ conversion went from 79 to 84.9%, while CO_2_ conversion went from 63 to 68.1%. These enhancements were attributed to smaller metal particle size and strong interaction between the metal and the support, facilitating the transport of the reactant. The influence of chromium was also associated with improvements in methane decomposition. Rastegarpanah et al. [62], when studying Ni/MgO catalysts for catalytic methane decomposition, found that by adding 10% of Cr to the catalyst, CH_4_ conversion increased by 10%. This increased activity was linked to Cr’s ability to provide a larger catalytic surface area and improve its reducibility. These findings suggest that chromium could have contributed to the effects observed in this research.

Tuan Abdullah and Croiset [37] demonstrated that the wall of the Inconel 625 reactor exhibited catalytic activity during the reforming of ethanol in supercritical water. This activity was attributed to the alloy’s high nickel content. A kinetic analysis confirmed that ethanol dehydrogenation primarily occurred via wall-catalyzed reactions on Inconel 625’s surface rather than through homogeneous reactions in the bulk fluid. Boukis et al. [63] studied the catalytic role of Inconel 625 for reforming methanol in supercritical water, and chromium-nickel oxides were found on the surface of the used reactor, accelerating the decomposition of methanol into carbon monoxide and hydrogen while also favoring the water–gas shift reaction.

Kim et al. [64] proposed a process called chemical looping dry reforming (CLDR) in which a reducible metal oxide acts as oxygen carrier and donates lattice oxygen to methane, partially oxidizing it into syngas (Equation (31)). At this junction, the metal oxide is reduced and, subsequently, CO_2_ causes the reduced oxygen carrier to be reoxidized, thus producing more CO (Equation (32)). NiFe_2_O_4_/Al_2_O_3_ was used as the oxygen carrier, showing conversions around 99% and 87% for CH_4_ and CO_2_, respectively.

(31) $${CH}_{4}+{MeO}_{x}\to CO+2H_{2}+{MeO}_{x-1}$$

(32) $${CO}_{2}+{MeO}_{x-1}\to CO+{MeO}_{x}$$

Yet, in Guan et al.’s study [65], NiO/Fe_2_O_3_ oxygen carriers were used for the CLDR process. It was found that NiO, in addition to Fe_2_O_3_, lowered the energy barrier for the rate-limiting step of methane dehydrogenation, improving their catalytic activity [65]. Fe_2_O_3_, MoO_2_, Cr_2_O_3_, CeO_2_, NiO, and Fe-Ni are some examples presented in the literature as oxygen carriers used specifically for CLDR processes.

Considering all of the metal oxides present in the Inconel reactor, it is plausible that they interacted with CH_4_ and CO_2_ to raise the production of CO and H_2_, as shown in Equations (31) and (32). Specifically, the Inconel reactor contained metal oxides such as nickel oxide, chromium oxide, chromium trioxide, chromium-nickel oxide and chromium iron oxide. These oxides, much like in a CLDR process, may have acted as effective oxygen carriers, contributing to the chemical looping mechanism. While the high nickel content in Inconel is certainly a key factor in its superior performance compared to that of the stainless steel (SS) reactor, the presence of chromium and iron oxides likely played an equally important role. Chromium oxides, known for their excellent reducibility and stability at high temperatures, could have enhanced the catalyst’s resistance to carbon deposition and promoted better CO_2_ conversion. Iron oxides, in turn, could have contributed to improved syngas production through their ability to facilitate redox cycling. The enhanced catalytic activity and stability of these metal oxides in the Inconel reactor led to a higher conversion rate of CH_4_ and CO_2_ into CO and H_2_ and improved overall reactor performance.

## 3. Materials and Methods

The primary syngas composition used in this work was an emulation of the output product from the supercritical water gasification (SCWG) of biomass derived from the experimental works from Karlsruhe Institute of Technology (KIT) as part of the CERESiS project. This gas consisted primarily of a high amount of H_2_, CO_2_ and CH_4_, almost no CO, and a small amount of C_2+_ alkanes (namely C_2_H_4_, C_2_H_6_, C_3_H_6_, C_3_H_8_) [9,66]. The emulated gas compositions are shown in Table 9.

**Figure 12:**
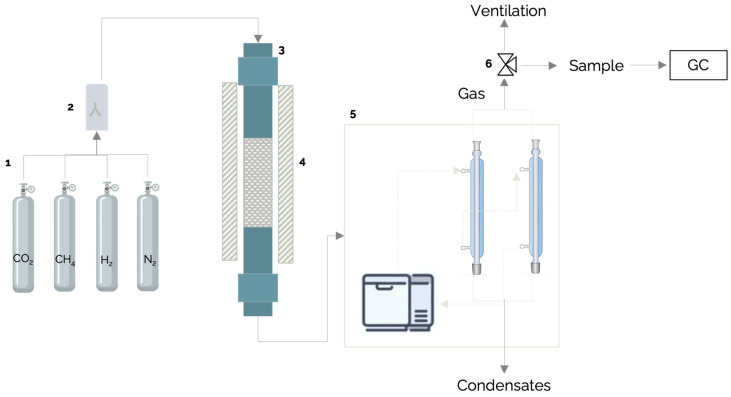
Simplified flow diagram of the reforming setup used in this work (**1**: individual gas cylinders for CO_2_, CH_4_, H_2,_ and N_2_; **2**: static mixer; **3**: fixed-bed reactor; **4**: oven; **5**: cold trap system, including a chiller and condensers; **6**: 3-way valve).

All experiments were performed using gas composition 2. Gas compositions 1, 3 and 4 were used only for one set of experiments, where the effect of the CH_4_/CO_2_ ratio on reforming was evaluated. The conditions originally tailored for dry reforming technologies were used to reform the gas. In this sense, CH_4_ and CO_2_ in the gas were expected to react and produce more CO and H_2_ (Equation (2)). A simplified flow diagram for the reforming setup used is shown in Figure 12.

As shown in Figure 12, each gas was stored in a compressed gas cylinder. To achieve the gas compositions outlined in Table 9, calibrated mass flow controllers regulated the desired volumetric flow for each gas (in mL/min) entering the reactor. In addition to H_2_, CH_4_, and CO_2_, nitrogen (N_2_) was used as an internal standard. The reactor employed in the experiment was a fixed-bed reactor. Two different material reactors were tested: one made of Inconel (alloy 625, Internal diameter (ID) = 2 in, height (H) = 48 in), and one made of stainless steel (SS) (ID = 1.84, H = 48 in). In addition to their composition materials, the difference was in the heating temperatures each could withstand. Moreover, knowing the probability of an RWGS reaction, a cold trap was placed right after the reactor outlet to ensure water condensation. During the experiment, the cold trap collected all produced water. The trap was emptied simultaneously when gas samples were taken. The weight of the water in grams was measured, and the time taken to reach that weight was also recorded. The water production rate in grams per hour (g/h) was calculated by dividing the weight in grams by the time in hours.

Reaction temperatures varied between 600 and 950 °C for the reactions carried out with the Inconel reactor, and from 600 to 800 °C for the SS reactions. The different operating temperature ranges were selected based on the inherent limitations of each material, with Inconel capable of withstanding up to 1095 °C and stainless steel up to 870 °C. An atmospheric pressure and gas velocity of 51.8 cm min^−1^ was applied for all the tests, and steel wool (SW) was employed for the catalyzed reactions. The steel wool used in this work was from the Bulldog^®^ brand (Thamesville, ON, Canada) and was composed of about 98% iron (Fe) [18].

Equation (33) was used to calculate the conversion of CH_4_ and CO_2_, where “i” is the compound in question, either CH_4_ or CO_2_.

(33) $$X_{\left(i\right)}\left(\%\right)=\frac{{{Flow}_{In}}_{\left(i\right)}-{{Flow}_{Out}}_{\left(i\right)}}{{{Flow}_{In}}_{\left(i\right)}}$$

The gas exiting the reactor was collected in a sampling bag (a 500 mL Tedlar^®^ bag with Polypropylene valve and septum fitting from Restek Corporation, Bellefonte, PA, USA) and then analyzed using a gas chromatograph (GC) (Bruker scion 456-GC, Bruker Corporation, Billerica, MA, USA). The GC system used for this purpose has three channels: two thermal conductivity detectors (TCD) and one flame ionization detector (FID). The first TCD channel was calibrated for H_2_, O_2_, N_2_, CH_4_ and CO detection, using two columns for gas separation, a Molsieve 13X, 80/100 mesh, 1.5 m × 1/8″ IS, and a Hayesep N, 80/100 mesh, 0.5 m × 1/8″ IS (both manufactured by Agilent Technologies, Santa Clara, CA, USA). The FID channel was used for longer chain hydrocarbon separation and detection and connected to a BR-1, 10 m × 0.15 mm, 2 µm column.

Energy-dispersive X-ray spectroscopy (EDS) was employed to ascertain the elemental composition of certain metals. Analyses were conducted utilizing an Electron Microscope (Desktop scanning electron microscope (SEM) equipped with EDS) from ThermoFisher Scientific Phenom XL G1 (Waltham, MA, USA). SEM was used for high-resolution imaging and detailed morphological insights. This allowed precise identification of surface features, guaranteeing that the analyzed zones would be representative of the sample. Combined with EDS, this can enable an in-depth study of the elemental distribution across the sample’s surface. Before analysis, the samples were prepared by embedding them in epoxy resin, followed by cutting and polishing until a very-low-roughness section was obtained. This step was essential for accurate mapping and quantitative analysis, as samples must be flat and smooth to minimize measurement errors. Quantitative analysis on a tilted or rough surface can lead to significant errors, potentially up to 100% for certain elements. Finally, a conductive metal coating (gold) was applied via sputter coating to prevent charging effects during analysis.

In the pursuit of precise crystalline phase identification, X-ray diffraction (XRD) analyses were conducted on select samples using the X’Pert Pro MPD diffractometer manufactured by Malvern Panalytical (Abingdon, Oxon, United Kingdom). A copper (Cu) Kα radiation source was employed, with a wavelength (λ) of 1.54187 angstroms (Å). The measurement range for 2θ spanned from 15° to 80. A step size of 0.04° was used to capture fine details in the diffraction patterns, enhancing analysis resolution. Each measurement was conducted over 260 s. The XRD experiments were conducted under stable operating conditions, with the instrument set to a voltage of 40 kilovolts (kV) and a current of 50 milliamperes (mA). The acquired diffraction data were processed and analyzed using the functionalities of JADE software (version 7.5.0, 2019) for peak identification, phase quantification, and structural analysis of the crystalline phases present in the samples.

## 4. Conclusions

In this work, an Inconel reactor exhibited better performances than the SS reactor when reforming primary syngas leading to the production of more H_2_ and CO while leaving less CH_4_ and CO_2_ in the reformed gas. This shows that the reactor’s material composition is crucial for improving reforming reactions. The high nickel and chromium content of the Inconel reactor made it intrinsically catalyst-like, allowing for higher conversion of CH_4_ and CO_2_. Conversely, the SS reactor’s exposition revealed another oxide distribution with less nickel content and poorer catalytic efficiency. Moreover, the effect of temperature rise was evident in the reaction. Higher temperatures increased conversions of CH_4_ and CO_2_, as expected for the endothermic dry reforming reaction with the highest conversion rates at 950 °C. The presence of steel wool in response to the Inconel reactor decreased CH_4_ and CO_2_ rates of conversion. This negative effect is probably due to the fact that steel wool disturbs the active catalytic sites or inhibits the generation of active species required for the reaction. The metal oxides formed inside the reactor can also contribute significantly to catalytic activity. Nickel oxide, chromium oxide, and chromium-nickel oxide were found to be present in the Inconel reactor by EDS and XRD analyses. These oxides probably acted as oxygen carriers that enhance the reforming process by allowing a better interaction between CH_4_ and CO_2_, probably increasing the productivity of CO and H_2_. Consequently, for the dry reforming of methane, the superior performance of the Inconel reactor is explained by the high nickel content and the formation of certain metal oxides, which enhance catalytic activity. These findings have shown how critical the composition of the reactor material is and how engineering can enable it to optimize reforming processes.

## Figures and Tables

**Table 1:** H_2_/CO ratio and water produced (in g/h) for reactions with and without SW in an Inconel reactor, at temperatures from 600 to 950 °C.

Temperature (°C)	Condition	H_2_/CO	Water Produced (g/h)
600	Without SW	1.67	3.79 ± 0.92
	With SW	1.84	3.26 ± 1.37
700	Without SW	1.63	4.06 ± 0.27
	With SW	1.32	3.62 ± 1.14
800	Without SW	1.38	1.85 ± 0.21
	With SW	1.04	4.46 ± 0.96
900	Without SW	1.28	1.74 ± 0.19
	With SW	1.30	2.04 ± 0.38
950	Without SW	1.33	1.65 ± 0.14
	With SW	1.28	2.17 ± 0.75

**Table 2:** CH_4_ and CO_2_ conversions after reforming the SCWG produced (Inconel reactor, temperature = 950 °C, without steel wool).

Gas Produced by SWCG with a Different Biomass	Molar CH_4_/CO_2_ Ratio Fed in the Process	Conversions (%)	
		CH_4_	CO_2_
1	0.66	100.00	86.82
2	0.62	100.00	81.69
3	0.47	100.00	75.94
4	0.52	98.84	78.20

**Table 3:** H_2_/CO ratio and water produced (in g/h) from reforming (without SW), in Inconel and SS reactors, at temperatures ranging from 600 to 800 °C.

Temperature (°C)	Reactor	H_2_/CO	Water Produced (g/h)
600	Inconel	1.67	3.79 ± 0.92
	Stainless Steel	4.86	0.75 ± 0.33
700	Inconel	1.63	4.06 ± 0.27
	Stainless Steel	1.46	4.25 ± 0.19
800	Inconel	1.38	1.85 ± 0.21
	Stainless Steel	0.92	4.39 ± 1.55

**Table 4:** Elemental concentration generated by EDS analysis of sample A (extracted from the Inconel reactor used in the experiments).

Element Name	Weight Concentration (%)
Oxygen	30.49
Nickel	49.46
Chromium	9.40
Iron	9.97
Silicon	0.23
Manganese	0.29
Titanium	0.16

**Table 5:** Elemental concentration generated by EDS analysis in two zones from sample A.

Element Name	Weight Concentration (%)	
	Zone 1	Zone 2
Oxygen	24.26	-
Nickel	56.46	70.15
Chromium	4.68	14.45
Iron	8.49	10.35
Silicon	0.23	-
Manganese	0.21	-
Carbon	5.68	5.04

**Table 6:** Elemental concentration generated by EDS analysis in two zones from sample B.

Element Name	Weight Concentration (%)	
	Zone 1	Zone 2
Oxygen	39.86	-
Nickel	1.48	80.91
Chromium	58.13	9.55
Iron	0.39	8.94
Titanium	0.14	-
Silicon	-	0.39
Aluminum	-	0.21

**Table 7:** Possible reactions that occurred on the Inconel 625 alloy to produce the several oxides found on samples A and B (adapted from S. Guo et al. [57]).

Reaction		Equation Number
Metal loss of electrons	$Ni\to {Ni}^{2+}+2e^{-}$	(10)
	$Cr\to {Cr}^{3+}+3e^{-}$	(11)
	$Fe\to {Fe}^{2+}+2e^{-}$	(12)
	$Fe\to {Fe}^{3+}+3e^{-}$	(13)
NiO formation	Ni2++12O2+2e−→NiO	(14)
	$Ni+H_{2}O↔NiO+2H^{+}+2e^{-}$	(15)
	$Ni+H_{2}O↔NiO+H_{2}$	(16)
Fe_3_O_4_ formation	$3Fe+{4H}_{2}O\to {Fe}_{3}O_{4}+4H_{2}$	(17)
	$3Fe+2O_{2}\to {Fe}_{3}O_{4}$	(18)
	${Fe}^{2+}+{Fe}^{3+}+4O^{2-}\to {Fe}_{3}O_{4}$	(19)
Cr_2_O_3_ formation	2Cr+32O2↔Cr2O3	(20)
	$2Cr+3H_{2}O\to {Cr}_{2}O_{3}+3H_{2}$	(21)
CrO_3_ formation	Cr+32O2↔CrO3	(22)
	Cr2O3+32O2↔2CrO3	(23)
Cr_2_NiO_4_ formation	$NiO+{Cr}_{2}O_{3}\to {Cr}_{2}{NiO}_{4}$	(24)
	$Ni+2Cr+4H_{2}O\to {Cr}_{2}{NiO}_{4}+2H_{2}$	(25)
	${Ni}^{2+}+2{OH}^{-}{+Cr}_{2}O_{3}\to {Cr}_{2}{NiO}_{4}+2H_{2}O$	(26)
Cr_2_FeO_4_ formation	${Cr}_{2}O_{3}+H_{2}O+Fe\to {Cr}_{2}{FeO}_{4}+H_{2}$	(27)
	${2Cr}_{2}O_{3}+O_{2}+2Fe\to {2Cr}_{2}{FeO}_{4}$	(28)
	${4Cr}_{2}O_{3}+{Fe}_{3}O_{4}+Fe\to {4Cr}_{2}{FeO}_{4}$	(29)
	${Fe}^{2+}+{Fe}^{3+}+{Cr}^{3+}+4O^{2-}\to {Cr}_{2}{FeO}_{4}$	(30)

**Table 8:** Elemental concentration generated by EDS analysis in 4 zones from a sample of SS reactor used in the reactions.

Element Name	Weight Concentration (%)			
	Zone 1	Zone 2	Zone 3	Zone 4
Iron	46.26	69.69	65.63	37.63
Oxygen	38.67	-	5.17	35.95
Chromium	5.96	16.68	15.70	16.87
Nickel	3.28	6.77	6.32	4.11
Carbon	3.11	4.58	5.06	2.80
Copper	1.06	-	-	-
Silicon	0.87	0.70	0.67	0.78
Manganese	0.79	1.58	1.45	0.93
Zinc	-	-	-	0.93

**Table 9:** Volumetric gas composition (%) used to emulate the product from the SCWG of different biomasses.

Gas	Biomass	H_2_ (vol.%)	CH_4_ (vol.%)	CO_2_ (vol.%)	CH_4_/CO_2_ Ratio
1	Reed Canary Grass (*Phalaris arundinacea*)	34.90	26.00	39.10	0.66
*2*	Switchgrass (*Panicum virgatum*)	33.05	25.73	41.23	0.62
3	Reed Canary Grass (*Phalaris arundinacea*)	34.00	21.00	45.00	0.47
4	Napier Grass (*Pennisetum purpureum*)	34.50	22.50	43.00	0.52

## Data Availability

The raw data supporting the conclusions of this article will be made available by the authors on request.
